# Recent Developed Strategies for Enhancing Chondrogenic Differentiation of MSC: Impact on MSC-Based Therapy for Cartilage Regeneration

**DOI:** 10.1155/2021/8830834

**Published:** 2021-03-20

**Authors:** Kangkang Zha, Zhiqiang Sun, Yu Yang, Mingxue Chen, Cangjiang Gao, Liwei Fu, Hao Li, Xiang Sui, Quanyi Guo, Shuyun Liu

**Affiliations:** ^1^Medical School of Chinese PLA, Beijing, China; ^2^Institute of Orthopaedics, Chinese PLA General Hospital; Beijing Key Lab of Regenerative Medicine in Orthopaedics, Key Laboratory of Musculoskeletal Trauma & War Injuries, PLA, 28 Fuxing Road, Haidian District, Beijing, China; ^3^School of Medicine, Nankai University, Tianjin, China; ^4^The Second People's Hospital of Guiyang, Guiyang, Guizhou, China; ^5^Department of Orthopaedic Surgery, Beijing Jishuitan Hospital, Fourth Clinical College of Peking University, Beijing, China

## Abstract

Articular cartilage is susceptible to damage, but its self-repair is hindered by its avascular nature. Traditional treatment methods are not able to achieve satisfactory repair effects, and the development of tissue engineering techniques has shed new light on cartilage regeneration. Mesenchymal stem cells (MSCs) are one of the most commonly used seed cells in cartilage tissue engineering. However, MSCs tend to lose their multipotency, and the composition and structure of cartilage-like tissues formed by MSCs are far from those of native cartilage. Thus, there is an urgent need to develop strategies that promote MSC chondrogenic differentiation to give rise to durable and phenotypically correct regenerated cartilage. This review provides an overview of recent advances in enhancement strategies for MSC chondrogenic differentiation, including optimization of bioactive factors, culture conditions, cell type selection, coculture, gene editing, scaffolds, and physical stimulation. This review will aid the further understanding of the MSC chondrogenic differentiation process and enable improvement of MSC-based cartilage tissue engineering.

## 1. Introduction

Articular cartilage damage is commonly seen in clinical practice and is often caused by trauma, progressive osteoarthritis (OA), and rheumatoid arthritis (RA). Due to its avascular nature, it is difficult for articular cartilage to undergo self-healing [1]. At present, common methods used for articular cartilage regeneration are microfracture [2], particulated articular cartilage implantation [3], osteochondral allograft or autograft transplantation [4, 5], and autologous chondrocyte implantation [6]. However, these techniques are limited in their ability to form hyaline cartilage. The development of cartilage tissue engineering strategies over the past few decades has provided a new approach for cartilage regeneration, which consists of three elements: seed cells, scaffolds, and growth factors [7].

Among various cell types, mesenchymal stem cells (MSCs) are one of the most promising seed cells for cartilage tissue engineering. MSCs are pluripotent adult stem cells that exhibit self-renewal, multipotent differentiation, and immunomodulation functions [8]. The International Society for Cellular Therapy has proposed the following standard criteria for MSCs: (1) MSCs must be plastic adherent in standard culture conditions; (2) MSCs must express CD105, CD73, and CD90 and not express CD45, CD34, CD14 or CD11b, CD79*α* or CD19, and HLA-DR; and (3) MSCs must be able to differentiate into osteoblasts, chondroblasts, and adipocytes *in vitro* [9]. A large number of basic studies and clinical trials employing MSCs for articular cartilage regeneration have been reported. Intra-articular injection of MSCs has been proven to be safe and effective for improving patients' pain, symptoms, and quality of life [10].

However, MSCs tend to lose their cellular functions, including their self-renewal ability and multipotency, after isolation and *in vitro* expansion, which could in part explain the treatment failures of several MSC-based clinical trials [11]. Numerous studies have indicated that under specific conditions, MSCs can form cartilage-like tissues that contain a certain amount of typical cartilaginous biomolecules, such as type II collagen (COL II), proteoglycans, and aggrecan. However, the composition and structure of the resulting differentiated tissues rarely reach the level of native cartilage. It has been proposed that the collagen content in tissue-engineered cartilage is generally less than 50% of that in native cartilage. In addition, the stratified ultrastructure and spatial organization of native cartilage is often not seen in tissue-engineered cartilage, which results in unsatisfactory mechanical properties [12]. Therefore, differentiating MSCs into normal chondrocytes and maintaining their physiological function are goals that need to be achieved in the field of cartilage regeneration. The regulation of MSC chondrogenic differentiation represents an area that has attracted an enormous amount of research, which is favorable for further understanding of the chondrogenic differentiation process and the optimization of MSC-based cartilage regenerative strategies [13].

In this review, we described the chondrogenic differentiation process of MSCs and then summarized the recent advances in enhancement strategies for MSC chondrogenic differentiation, including optimization of bioactive factors (Table 1), culture conditions, cell type selection, coculture, gene editing, scaffolds, and physical stimulation (Table 2). This review will help to improve the therapeutic effect of MSC-based therapy for cartilage regeneration.

## 2. Chondrogenic Differentiation Process of MSCs

**Figure 1:**
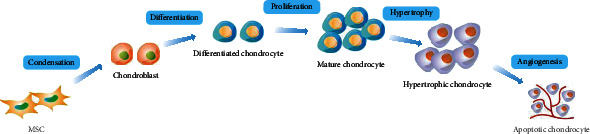
Chondrogenic differentiation process of mesenchymal stem cells (MSCs). The chondrogenic differentiation of MSCs is proposed to follow an endochondral ossification procedure, which includes five main stages: condensation, differentiation, proliferation, hypertrophy, and angiogenesis.

The cartilage is a connective tissue that is composed of chondrocytes and their surrounding matrix, which mainly contains collagens and proteoglycans. Chondrogenesis, the formation of chondrocytes and cartilage tissues, leads to the development of the various types of cartilage, including hyaline, fibrous, and elastic cartilages [14]. MSCs possess multipotent differentiation potential and can differentiate into numerous mesodermal cell types, such as chondrocytes, osteoblasts, adipocytes, and myofibroblasts [15]. In the process of chondrogenic differentiation, MSCs are thought to follow an endochondral ossification procedure, which includes five main stages (Figure 1). First, in the presence of certain paracrine factors, MSCs produce extracellular matrix (ECM) containing hyaluronan, collagen type I (COL I), and COL II and then undergo increased condensation through cell-ECM and cell-cell interactions. Second, MSCs differentiate into chondrocytes under the influence of a branch of transcription factors, such as Smads, p38, RhoA/ROCK, and SOX9. Third, differentiated chondrocytes proliferate rapidly and secrete ECM. Fourth, mature chondrocytes take on a hypertrophic phenotype and begin to express collagen type X (COL X) and alkaline phosphatase. Fifth, hypertrophic chondrocytes are replaced with blood vessels after cell death [14].

**Figure 2:**
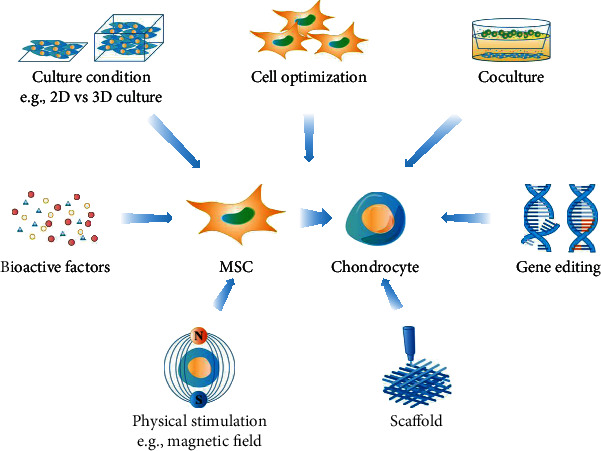
Approaches for enhancing MSC chondrogenic differentiation. Several methods have proven to be effective in promoting chondrogenic differentiation of MSCs, including optimization of bioactive factors, culture conditions, cell type selection, coculture, gene editing, scaffolds, and physical stimulation.

The differentiation of MSCs into chondrocytes requires a dynamic balance of various promoters and inhibitors. The microenvironment consists of soluble cytokines, surrounding matrix, nearby cells, and physical stimuli, all of which play an important role in determining the cellular fates and chondrogenic differentiation of MSCs (Figure 2). However, after differentiating into mature chondrocytes, MSCs may undergo cellular hypertrophy followed by vascular penetration, marrow deposition, and ossification. Exploring potential methods to inhibit unexpected chondrocyte hypertrophy and osteogenic differentiation could help to maintain the phenotype of mature chondrocytes differentiated from MSCs.

## 3. Bioactive Factors

### 3.1. Cytokines

Among the multiple cytokines required for initiating MSC chondrogenic differentiation, transforming growth factor beta (TGF-*β*) is the most commonly used [16]. TGF-*β* exists in three isoforms, TGF-*β*1, TGF-*β*2, and TGF-*β*3, and has two receptors, TGF-*β* receptors I (TGF-*β* RI) and II (TGF-*β* RII). After binding with TGF-*β* RI or TGF-*β* RII, TGF-*β* induces MSC chondrogenic differentiation mainly through the activation of the TGF-*β*/Smad signaling pathway. Phosphorylated Smad2/3 binds to Smad4 and translocates into the nucleus, resulting in the expression of *SOX9* and *COL II* [17]. Xu et al. indicated that the activation of RhoA/ROCK was also involved in TGF-*β*-induced chondrogenic differentiation of rat synovium-derived MSCs (SDSCs) through interaction with the Smad pathway [18]. MAPK signaling is another pathway through which TGF-*β* regulates MSC chondrogenic differentiation, and in this pathway, p38 promotes chondrogenic differentiation of human bone marrow-derived mesenchymal stem cells (BMSCs), while ERK-1 suppresses BMSC chondrogenic differentiation [19]. Bone morphogenetic proteins (BMPs) are members of the TGF-*β* superfamily and also participate in regulating human BMSC (hBMSC) chondrogenic differentiation. Among BMPs, BMP2, BMP4, BMP6, and BMP7 are the most widely employed for BMSC chondrogenic differentiation [20, 21].

In addition to TGF-*β* and BMPs, other cytokines have also been shown to enhance MSC chondrogenic differentiation. For example, Hagmann et al. revealed that the addition of fibroblast growth factor-2 (FGF-2) during the *in vitro* expansion of hBMSCs significantly enhanced their chondrogenic differentiation with no influence on their adipogenic or osteogenic differentiation [22]. Jeong et al. found that thrombospondin-2 not only promoted the chondrogenic differentiation of the human umbilical cord blood-derived mesenchymal stem cells (UCBSCs) through the activation of the Notch signaling pathway but also attenuated their hypertrophic differentiation [23].

### 3.2. KGN

Although cytokines play vital roles in inducing MSC chondrogenic differentiation, their applications may be restricted due to their short half-life and high cost. Recently, some small molecules have been found to enhance MSC chondrogenic differentiation, and these molecules are particularly intriguing because of their stability and low cost [24]. Kartogenin (KGN), first discovered by Johnson in 2012 [25], is an important small molecule that facilitates MSC chondrogenic differentiation and has drawn considerable interest in recent years [26–28]. Compared with TGF-*β*, KGN seems to induce a weaker promotion of chondrogenic differentiation but a greater suppression of chondrocyte hypertrophy in human adipose tissue-derived MSCs (ADSCs) [29, 30]. In addition, the combination of KGN and TGF-*β*3 has synergistic effects, as human umbilical cord-derived mesenchymal stem cells (UCMSCs) treated with KGN and TGF-*β*3 were shown to secrete more COL II than MSCs treated with TGF-*β*3 or KGN alone [31]. Zhou et al. reported that KGN can induce the differentiation of human SDSCs (hSDSCs) into chondrocytes through the activation of the BMP-7/Smad5 signaling pathway [32]. In addition, Jing et al. revealed that human UCMSCs (hUCMSCs) preconditioned with KGN were stalled in a precartilaginous stage with the activation of JNK/RUNX1 pathway and suppression of *β*-catenin/RUNX2 pathway [33]. After induction of chondrogenic differentiation by KGN, hBMSCs expressed significantly increased expression levels of *SERPINA9* and *SERPINB2*, which may serve as novel differentiation markers for MSC lineage commitment toward cartilage [34]. Several biomaterials have been synthesized to improve MSC chondrogenic differentiation through controlled release of KGN. For example, Sun et al. developed a collagen/chitosan/hyaluronic acid (HA) scaffold containing poly(lactide-co-glycolide) (PLGA) microspheres for controlled KGN release and cartilage regeneration [35]. Chen et al. fabricated a KGN-conjugated poly(ether-ester-urethane)urea scaffold and demonstrated that KGN on the scaffold could undergo stable sustained release, thus enhancing chondrogenic differentiation of hUCMSCs *in vitro* and cartilage regeneration in rabbits [36].

### 3.3. Melatonin

Melatonin (N-acetyl-5-methoxytryptamine) is an indolamine that was first isolated from the pineal tissue in 1957 [37]. In addition to participating in the modulation of various physiological functions, such as sleep, circadian rhythms, and neuroendocrine processes, recent studies have suggested that melatonin also plays an important role in regulating MSC differentiation [38, 39]. It has been proven that melatonin enhances hBMSC osteogenic and chondrogenic differentiation while inhibiting adipogenic differentiation [40, 41]. Gao et al. performed a study in which they induced hBMSC chondrogenic differentiation with chondrogenic medium containing vehicle or melatonin. They found that the synthesis of glycosaminoglycans (GAGs) and COL II and the gene expression levels of *ACAN*, *COL II*, and *SOX9* were higher in the melatonin group than in the control group. Furthermore, they confirmed that melatonin receptors were expressed on chondrogenic BMSCs. After treatment with a melatonin receptor antagonist, the effect of melatonin on the chondrogenic differentiation of BMSCs was blocked, indicating that melatonin promoted BMSC chondrogenic differentiation at least partially through melatonin receptors [42].

### 3.4. Chondroitin Sulfate

Chondroitin sulfate (CS), a type of GAG in connective tissues, has shown the capacity to enhance MSC chondrogenic differentiation by providing a chondroinductive microenvironment [43, 44]. Compared with poly(ethylene glycol) hydrogels, CS-based hydrogels are able to promote both chondrocyte-specific gene expression and cartilage ECM accumulation. Furthermore, CS can inhibit the hypertrophic differentiation of goat BMSCs, as evidenced by significantly downregulated expression of *COL X* [45, 46]. The stiffness of the hydrogels also has an impact on the function of CS. CS-containing hydrogels with low mechanical stiffness were reported to lead to more neocartilage deposition than those with high stiffness [47]. CS supplementation has been utilized as a biochemical cue in integrated cartilage tissue engineering. Moura et al. developed 3D porous poly(*ε*-caprolactone) scaffolds with CS supplementation, which were able to promote hBMSC proliferation, migration, and chondrogenic differentiation [48]. Similarly, Huang et al. fabricated an alginate foam scaffold supplemented with CS and found increased amounts of a cartilage-specific matrix in differentiated hBMSC cultures supplemented with CS than in those supplemented with CS-free foams [49].

### 3.5. Other Factors

In addition to the above bioactive factors, other factors modulating MSC chondrogenic differentiation have also been investigated. Fan et al. demonstrated that ghrelin, also called the “hunger hormone,” significantly promoted rat BMSC chondrogenic differentiation, as evidenced by the upregulated expression of *COL II*, *SOX9*, and *ACAN* and enhanced accumulation of collagen and GAGs *in vitro*, which may be related to increased intracellular phosphorylation of ERK1/2 and DNMT3A. Furthermore, delivery of ghrelin and TGF-*β*3 significantly improved the cartilage repair effect of BMSCs in rats compared with delivery of TGF-*β*3 alone [50]. In addition, Li et al. reported that atractylenolides, a traditional Chinese medicine, was able to promote rat BMSC chondrogenic differentiation via activation of the Sonic Hedgehog (SHH) signaling pathway [51]. Follistatin-like protein-1 (FSTL-1), an acidic cysteine-rich glycoprotein, also plays a role in regulating MSC chondrogenic differentiation [52]. FSTL-1-deficient mouse embryonic skull-derived MSCs exhibited significantly downregulated gene expression of *COL2A1* and *SOX9*, reduced ECM production, and decreased activity of the TGF-*β* signaling pathway [53].

## 4. Culture Conditions

MSCs tend to lose their differentiation potential as a result of culture stress or cell senescence when expanded *in vitro*. Articular cartilage resides at low oxygen tension (1-4% oxygen) *in vivo* [54]. The impact of hypoxia on MSC chondrogenic differentiation has been of particular interest. It was demonstrated that MSCs cultured under low oxygen tension exhibited enhanced early chondrogenic differentiation and reduced hypertrophic differentiation, as evidenced by higher expression levels of the chondrogenic markers *COL II*, *SOX9*, and *ACAN* and lower expression levels of the hypertrophic markers *COL X* and *MMP13* [55–57]. Portron et al. investigated the related intracellular mechanism and confirmed that low oxygen tension increased the DNA-binding activities of two biological effectors, HIF-1*α* and HIF-2*α*, which have been reported to be promoters of human ADSC (hADSC) chondrogenic differentiation [55]. In addition, recent studies of cartilage tissue engineering have investigated the effect of 3D culture on MSC chondrogenic differentiation, which represents a potential way to mimic the *in vivo* cartilage tissue environment. Synthetic and natural materials, such as 3D-printed bioreactor chambers, hydrogels, and microspheres, have been developed as tools to create a 3D microenvironment for MSCs [58–61]. For example, Sulaiman et al. compared the 2D and 3D cultures of hBMSCs and found that 3D culture of BMSCs on gelatin microspheres enhanced their stemness and chondrogenic differentiation compared to 2D culture on a standard tissue culture plate [61].

## 5. Cell Types

In recent years, many researchers have proposed that MSCs are heterogeneous and that not all share the same chondrogenic differentiation abilities. This heterogeneity was reported to exist among different donors, tissue sources, and cell phenotypes.

Among the various donor characteristics, the effect of donor age on MSC chondrogenic differentiation ability has been most frequently studied. Kanawa et al. isolated BMSCs from 17 patients (25-81 years old) and expanded them with FGF-2 for 28-42 days before differentiation assays. After 28 days of induced culturing, they found that the chondrogenic potential, rather than the osteogenic or adipogenic potential, of BMSCs declines with donor age, as evidenced by decreases in the expression of chondrocyte-specific genes such as *SOX9*, *COL2A*, and *ACAN*. Moreover, the (GAGs)/DNA content also significantly decreased with donor age after chondrogenic differentiation [62]. However, Andrzejewska et al. indicated that the chondrogenic potential of BMSCs was not affected by donor age. They examined the phenotypic and functional performances of BMSCs isolated from adult and elderly patients (*n* =10 and *n* =13, mean age 38 and 72 years old) and found no difference in proteoglycan synthesis between BMSCs (at passage 6) from younger adults and those from older adults after 21 days of chondrogenic differentiation induction [63]. Thus, it is still not clear whether MSC chondrogenic differentiation is affected by donor age, and further studies are needed. On the other hand, Dudics et al. demonstrated that the chondrogenic differentiation ability of BMSCs from OA and RA patients was comparable to that of BMSCs from healthy individuals, as shown by similar *COL II* gene expression and proteoglycan synthesis after chondrogenic induction, suggesting that BMSCs from OA and RA patients could also be applied in cartilage tissue engineering [64]. Garcı'a-A'lvarez reached a similar conclusion when they found that the chondrogenic differentiation potential of BMSCs from OA patients was similar to that of BMSCs from femoral fracture patients [65].

Conversely, it is well recognized that MSCs from different tissue sources possess different potentials for chondrogenic differentiation. Compared with BMSCs, ADSCs appear to have lower chondrogenic potential [66–68]. MSCs have also been identified in the synovial tissue, a tissue type that is adjacent to articular cartilage. SDSCs have shown higher chondrogenic potential than BMSCs and ADSCs [69]. However, Neybecker revealed that the chondrogenic differentiation potential of SDSCs was lower than that of BMSCs in advanced OA patients, which may be attributed to the intraarticular inflammatory environment caused by OA [70]. It has also been proposed that the chondrogenic differentiation and ECM production capacities of human amnion- and placenta-derived MSCs are higher than those of hADSCs [71, 72]. The chondrogenic differentiation potential of MSCs derived from the same tissue in different parts of the body also varies. For example, compared with those isolated from the femoral head bone marrow, hBMSCs isolated from the iliac crest and vertebral body bone marrow were more likely to differentiate into chondrocytes and form cartilaginous tissue *in vitro* [73].

MSCs from the same tissue are different in cellular phenotype. CD105^+^ SDSCs possess greater chondrogenic potential than CD105^−^ SDSCs. The promotion of SDSC chondrogenic differentiation by CD105 is achieved through the activation of the TGF-*β*/Smad2 signaling pathway [74–76]. Hagmann et al. revealed that after chondrogenic differentiation, CD146^+^ hBMSCs produced more GAGs than unsorted BMSCs [77]. Compared with CD106^+^ or CD73^+^ hSDSCs, CD271^+^ SDSCs exhibited a greater chondrogenic differentiation capacity, as determined by histological and immunohistochemical analyses for COL II [78]. Single-cell RNA sequencing (scRNA-seq) technology can be used to analyze gene expression at the single-cell level, enabling the identification of functional cell subpopulations, making it a powerful tool for investigating MSC heterogeneity [79]. Freeman et al. used scRNA-seq to assess the transcriptional diversity of mouse BMSCs and found that the expression of genes associated with multilineage potential and immunomodulation ability was inconsistent between individual cells [80]. Sun et al. investigated the gene expression profile of human Wharton's jelly MSCs (WJMSCs) via scRNA-seq and found some highly variable genes to be associated with the functional properties of WJMSCs. They found that different subpopulations showed distinct chondrogenic differentiation potency [81]. By performing scRNA-seq of the transcriptome, Liu et al. identified 3 subpopulations within hBMSCs, among which one subpopulation exhibited a strong expression of FGFR2 and potentially included skeletal stem cells [82]. Specifically, Merrick et al. demonstrated that dipeptidyl peptidase-4/CD26^+^ ADSCs represent highly proliferative and multipotent progenitors in murine and human adipose tissues, while their chondrogenic differentiation ability still needs further investigation [83]. Additional research is needed to explore more functional MSC subpopulations via scRNA-seq to identify those with greater chondrogenic differentiation potential.

## 6. Coculture

Coculture was first performed in 1978 by Lawrence et al., who indicated that heterologous cells communicated and responded to cell-specific hormones through cyclic AMP [84]. In recent years, coculture has been applied in cartilage tissue engineering [85]. It was reported that the presence of chondrocytes promoted MSC chondrogenic differentiation in culture [86, 87]. Compared to direct coculture, indirect coculture with human UCBSCs and chondrocytes significantly increased the expression of *SOX9* and *COL II* and decreased the expression of *COL I* in UCBSCs [88]. Kubosch et al. revealed that coculture of human or swine SDSCs with chondrocytes resulted in greater self-organization, chondrogenic differentiation, and TGF-*β* secretion in SDSCs, suggesting that chondrocytes may induce a chondrogenic phenotype in SDSCs through paracrine action mimicking joint homeostasis [89, 90]. *In vivo* ectopic chondrogenic differentiation of swine BMSCs could also be induced by mature chondrocytes, which may be attributed to soluble chondrogenic factors secreted by chondrocytes [91]. In addition, when cocultured with hADSCs, chondrocytes were shown to suppress the undesired hypertrophy of hADSCs [92]. Zhang et al. carried out a study in which human WJMSCs and chondrocytes were cocultured on an acellular cartilage ECM scaffold and transplanted into the articular cartilage defect area in caprine. After 9 months, they found that the neotissue was more similar to native cartilage than that formed by the transplantation of WJMSCs or chondrocytes alone, indicating that coculture represents a promising strategy for improving the cartilage-regenerating effects of MSCs [93]. However, to determine the optimal culture conditions, MSC and chondrocyte cocultures need to be further investigated in more *in vivo* models. In addition, the impact of coculturing MSCs with other cell types on MSC chondrogenic differentiation should also be evaluated [94].

## 7. Gene Editing

The overexpression and knockdown of specific genes are optional methods to control chondrogenic differentiation in MSCs. *DLX5* is a member of the *DLX* gene family, and DLX5 associates with HOXC8 to form a protein complex. Yang et al. revealed that the expression of both *DLX5* and *HOXC8* was increased during chondrogenic differentiation of human apical papillae-derived MSCs (APSCs) and that the overexpression of *DLX5* and *HOXC8* promoted the chondrogenic differentiation of APSCs. In fact, the protein complex formed by *DLX5* and *HOXC8* could inhibit the activation of *LINC01013*, a negative regulator of chondrogenesis, by directly binding to its promoter [95]. Similarly, *KLF15*, a member of the *KFL* transcription factor family, is also upregulated when hBMSCs undergo chondrogenic differentiation. By binding to the *SOX9* promoter, *KFL15* was shown to activate *SOX9* and enhance the chondrogenic differentiation potential of BMSCs [96]. In addition, Zhou et al. found that *corin* expression was upregulated in the trilineage differentiation process of hBMSCs. The silencing of *corin* gene expression inhibited chondrogenic (rather than osteogenic and adipogenic) differentiation of BMSCs, indicating that *corin* may play a positive role in the regulation of chondrogenic differentiation of BMSCs [97]. Tian et al. demonstrated that miR-30a also plays an important role in chondrogenic differentiation of rat BMSCs by inhibiting *DLL4* expression [98]. In another study, Kim et al. fabricated *shATF4* and *SOX9* plasmid DNA complexed with gene regulation nanoparticles and verified that it could significantly promote the chondrogenic differentiation of hBMSCs [99]. In addition, it was demonstrated that *H-89* could increase miR-23b expression in human MSCs (hMSCs), thus promoting their chondrogenic differentiation through inhibition of PKA signaling [100]. All of these genes may be potential targets for gene editing to enhance MSC chondrogenic differentiation. However, the safety of gene editing in MSCs needs to be fully explored before this strategy can be applied clinically.

## 8. Scaffolds

Researchers are constantly attempting to fabricate scaffolds that are able to enhance MSC chondrogenic differentiation. It has been proposed that the physical properties of the scaffolds are involved in regulating MSC chondrogenic differentiation. Ahmed et al. developed 16 electrospun scaffolds with different stiffness and wettability and revealed that chondrogenic differentiation of ATDC5 cells were enhanced in soft scaffolds with an intermediate wettability as evidenced by an increased level of cartilage-associated gene expression [101]. In another study, Nalluri et al. synthesized a hydrophilic polyurethane scaffold with gel like architecture and found that it enhanced BMSC chondrogenic differentiation, as determined by significantly increased cartilage-specific ECM production [102]. Additionally, the porosity and pore size of scaffolds also play a role in MSC chondrogenic differentiation. Prasopthum et al. demonstrated that 3D-printed scaffolds with micro/nanoporous structures could promote chondrogenic and osteogenic differentiation of hBMSCs better than scaffolds with nonporous structures [103]. It was reported that small-pore scaffolds (pore size of 125-250 *μ*m) were more likely to enhance chondrogenic differentiation and inhibit endochondral ossification of hBMSCs compared with large-pore scaffolds (pore size of 425-600 *μ*m) [104]. Interestingly, Di Luca et al. created scaffolds composed of poly(ethylene oxide therephtalate)/poly(butylene therephtalate) with a structural gradient in pore size. They confirmed that hBMSCs seeded on the gradient scaffolds produced more GAGs as compared with those seeded on nongradient scaffolds [105].

As a biologically complete substrate, ECM has been proposed to provide a native microenvironment for MSCs and to aid in the maintenance of their functions [106, 107]. Coating with ECM has been shown to preserve the stemness and differentiation potential of *in vitro*-expanded MSCs [108]. Compared with polyglycolic acid (PGA) scaffolds, ECM scaffolds not only enhanced chondrogenic differentiation of rabbit BMSCs more effectively but also maintained the BMSC phenotype for longer *in vivo* [109]. Li et al. demonstrated that cartilage ECM could not only enhance chondrogenic differentiation but also inhibit hypertrophic differentiation of hBMSCs. Among various ECM collagen subtypes, collagen type XI exhibited the strongest effects on promoting the production and inhibiting the degradation of cartilage matrix [110]. Collagen and GAGs are ideal natural materials that can mimic the matrix niche of chondrocytes and reportedly have an enhancing effect on the chondrogenic differentiation of MSCs [111]. Raghothaman et al. fabricated an interfacial polyelectrolyte complexation-Col I hydrogel and found that it could enhance cell-cell interactions and cellular condensation, thereby resulting in improved hBMSC chondrogenic differentiation and hyaline neocartilage formation [112]. In another study, Meng et al. generated a tricalcium phosphate-collagen-hyaluronan scaffold and found that it efficiently induced chondrogenic differentiation of ATDC-5 cells and hBMSCs without the need for exogenous growth factors [113]. Similarly, Moulisová et al. constructed a gelatin-HA hybrid hydrogel and confirmed that it promoted both chondrogenic differentiation and adhesion of hBMSCs [114]. Feng et al. synthesized sulfated HA hydrogels and found that they not only promoted MSC chondrogenic differentiation but also suppressed hMSC hypertrophy. When utilized to treat OA in rats, the sulfated HA hydrogels significantly reduced cartilage abrasion and hypertrophy [115].

Additionally, previous works have shown that biomaterials can be used as effective delivery vehicles or bioactive matrices to promote MSC chondrogenic differentiation and mitigate MSC hypertrophy. Morille et al. generated PLGA-based microspheres coated with TGF-*β*3 and confirmed their promotion of chondrogenic differentiation of MSCs *in vitro*. When hBMSCs seeded onto these microspheres were injected into the knee cavities of rats with OA, cartilage-like tissue was formed, and decreased degradation of endogenous articular cartilage was observed after 6 weeks [116]. In addition, Xu et al. fabricated a multifunctional nanocarrier modified with RGD peptide and *β*-cyclodextrin that could carry siRNA targeting Runx2 and small molecules such as KGN. They verified that it was able to induce hMSC differentiation into chondrocytes and suppress their hypertrophy [117]. Remote control of MSC chondrogenic differentiation *in vivo* via biomaterials has also been achieved. Based on an upconversion nanotransducer, Kang et al. developed a nanocomplex with photolabile caging of KGN and calcium, whose release could be triggered by near-infrared light. They confirmed that intracellular KGN and calcium delivery promoted chondrogenic differentiation and inhibited the hypertrophy of hMSCs *in vivo* [118].

## 9. Physical Stimulation

### 9.1. Mechanical Stimulation

Articular cartilage is a smooth wear-resistant connective tissue that can withstand complex mechanical stimuli and distribute loads to the subchondral bone. Proper mechanical stimulation has been revealed to upregulate the gene expression of *ACAN* and *COL II* in chondrocytes while maintaining their phenotypes, thus promoting cartilage formation [119–121]. Similarly, an in-depth understanding of the effect of mechanical stimulation on MSC chondrogenic differentiation may facilitate the success of MSC-based cartilage regenerative therapies in joints, which have a mechanically demanding environment. It is proposed that MSCs respond to mechanical stimulation through autocrine or paracrine activity to enhance their chondrogenic differentiation and capacity for repairing cartilage damage. Various types of mechanical stimulation have been applied to enhance MSC chondrogenic differentiation in cartilage tissue engineering [122]. Hou et al. demonstrated that low-magnitude high-frequency vibration enhanced the chondrogenic potential of rat BMSCs through activation of the Wnt/*β*-catenin signaling pathway [123]. Xie et al. revealed that proper tensile mechanical stimulation could improve the viscoelasticity and chondrogenic phenotype of rabbit BMSCs [124]. Additionally, Zhang et al. investigated the effect of deferral dynamic compression on the chondrogenic differentiation of hBMSCs and found that it enhanced chondrogenic differentiation and suppressed chondrocyte hypertrophy, accompanied by the activation of TGF-*β*/Activin/Nodal signaling pathway and suppression of BMP/GDP and integrin/FAK/ERK signaling pathways [125]. Cao et al. performed a similar study in which they applied dynamic mechanical loading to rabbit BMSCs-collagen scaffold constructs and found that BMSCs expressed higher levels of *ACAN*, *COL2A1*, and *SOX9* and lower levels of *COL10A1* and *COL1A2*. The mechanical strength of the constructs was significantly improved and was similar to that of native cartilage [126]. Indian Hedgehog (IHH) and SHH can promote MSC chondrogenic differentiation but tend to result in chondrogenic hypertrophy and ossification. Chen et al. reported that microgravity caused by a rotary cell culture system was able to enhance chondrogenic differentiation of rabbit BMSCs while attenuating the chondrocyte hypertrophy and aging induced by IHH and SHH [28].

In addition, recent studies have demonstrated that low-intensity pulsed ultrasound (LIPUS), which provides mechanical stimulation in the form of sound waves, can be used to promote chondrogenic differentiation of C3H10T1/2 cells [127]. After LIPUS stimulation at 3 MHz, BMSCs secreted increased amounts of cartilage-like ECM and showed upregulated expression of chondrogenic genes, such as *COL II*, *SOX9*, and *ACAN*. The stimulatory effect of LIPUS on rat BMSC chondrogenic differentiation is reportedly achieved through inhibition of autography [128]. Cui et al. seeded rabbit BMSCs on a PGA scaffold and implanted the construct into the backs of nude mice, which subsequently received LIPUS stimulation for 10 min every day for 4 weeks. They found that the collagen and GAG content, as well as the mechanical properties, showed a more significant increase in the LIPUS group than in the unstimulated group, suggesting that LIPUS stimulation could promote BMSC chondrogenic differentiation *in vivo* [129].

### 9.2. Electric Field

In addition to mechanical stimulation, other physical stimuli, such as electrical and electromagnetic/magnetic stimuli, also have an impact on the chondrogenic differentiation of MSCs [130]. Treatment with a low-frequency electric field (EF) was reported to result in increased expression of *COL II* and *SOX9* and decreased expression of *COL I* and *COL X* in hADSCs [131, 132]. Even in the absence of exogenous growth factors, a low-frequency EF could enhance chondrogenic differentiation of mouse BMSCs. It was demonstrated that EF promoted BMSC chondrogenic differentiation by driving Ca^2+^/ATP oscillations, which are known to play an important role in prechondrogenic condensation. In addition, EF was found to induce increased TGF-*β*1 expression, and the inhibition of TGF-*β* signaling blocked EF-driven BMSC chondrogenic differentiation, indicating that TGF-*β* signaling mediates EF-driven BMSC chondrogenic differentiation. Other signaling pathways, including BMP signaling and MAPK signaling, have also been proposed to be involved in regulating the effect of EF treatment on BMSC chondrogenic differentiation [133, 134]. Additionally, Li et al. revealed that nanosecond pulsed EF (nsPEF) downregulated the expression of *DMMT1*, thus increasing the methylation of the *OCT4* and *NANOG* promotors. As a result, swine BMSCs treated with nsPEF exhibited enhanced trilineage differentiation ability [135].

### 9.3. Electromagnetic Field

Electromagnetic field (EMF) has also been shown to promote MSC chondrogenic differentiation [136]. Mayer-Wagner et al. investigated the impact of EMF on hBMSCs during chondrogenic differentiation and found that BMSCs exposed to a low-frequency EMF (5 mT) showed higher *COL II* expression, increased (GAGs)/DNA content, and lower *COL X* expression than those that had not been treated with an EMF [137]. Analogously, Parate et al. demonstrated that optimal hBMSC chondrogenic differentiation was achieved with a brief (10 min), low-intensity (2 mT) pulsed EMF exposure before chondrogenic induction rather than prolonged and repetitive EMF exposure. Transient receptor potential channels, a conduit for extracellular calcium, might be involved in mediating pulse EMF-driven BMSC chondrogenic differentiation [138].

## 10. Conclusions and Perspectives

MSCs have shown great prospects in cartilage tissue engineering. However, some issues need to be resolved before they can be widely applied. First, MSC-based therapy is largely limited by the ability to obtain and manufacture applicable MSC products because MSCs expanded *in vitro* are prone to losing their therapeutic potential and safety attributes [139]. Developing strategies to enhance chondrogenic differentiation in MSCs is necessary and has important clinical value for cartilage regeneration. In the present review, we summarized the recent research progress in MSC chondrogenic differentiation modulation, including optimization of bioactive factors, culture conditions, cell type selection, coculture, gene editing, scaffolds, and physical stimulation. Although all of these methods are effective in regulating chondrogenic differentiation of MSCs, the reliability, safety, and degree of difficulty in implementing these methods need to be considered. Second, because MSCs tend to undergo hypertrophy in their chondrogenic differentiation process, it is difficult for them to form hyaline cartilage *in vivo* [12]. A more comprehensive understanding of embryonic chondrogenesis would be beneficial for guiding MSCs to differentiate into cells with a cartilage phenotype. It has been suggested that MSC chondrogenic differentiation may occur in two different directions: one leading to bone formation via endochondral ossification and the other leading to articular cartilage formation. Although endochondral ossification has been widely used as a model to establish MSC chondrogenic differentiation protocols, chondrogenic differentiation of cartilage chondrocytes should be used instead to alleviate inevitable hypertrophic differentiation [140]. Third, the underlying mechanisms by which endogenous and transplanted MSC function remain to be elucidated. In-depth research has revealed that MSCs can perform a paracrine action and are capable of secreting diverse bioactive molecules, such as growth factors, cytokines, and chemokines [141, 142]. It is suggested that the chondrogenic differentiation of endogenous MSCs is involved in cartilage regeneration, but this is not necessarily true for implanted MSCs, which mainly work through immunomodulatory functions. To further improve the cartilage-regenerating ability of MSCs, additional strategies to recruit host MSCs and enhance their chondrogenic differentiation are still needed. It is also essential to exploit approaches to enhance MSC paracrine and immunomodulatory functions.

## Figures and Tables

**Table 1:** Effects of different bioactive factors on MSC chondrogenic differentiation.

Bioactive factors	Cell type	Signaling pathway	Dose	Effect	Ref.
TGF-*β*3	Human BMSCs	Activate TGF-*β*/Smad pathway	10 ng/mL	Promote MSC chondrogenic differentiation	[16, 17]
TGF-*β*1	Rat SDSCs	Activate RhoA/ROCK pathway and Smad pathway	10 ng/mL	Induce gene expression of *SOX9*, *COL I*, *COL II*, and *ACAN*	[18]
	Human BMSCs	Activate MAPK pathway and Wnt pathway	10 ng/mL	Induce gene expression of *SOX9*, *COL II*, and *ACAN* and proteoglycan synthesis	[19]
BMPs	Human ADSCs and BMSCs	NA	500 ng/mL	BMP-2, BMP-4, BMP-6, and BMP-7 are effective enhancers of MSC chondrogenic differentiation	[21]
FGF-2	Human BMSCs	NA	10 ng/mL	Increase GAG/DNA content	[22]
TSP-2	Human UCBSCs	Activate Notch pathway	NA	Promote the chondrogenic differentiation of MSCs and attenuate their hypertrophic differentiation	[23]
KGN	Human ADSCs	NA	100 nM/L	Promote chondrogenic differentiation and suppress chondrocyte hypertrophy in MSCs	[30]
	Human SDSCs	Activate BMP-7/Smad5 pathway	1 *μ*M/L and 10 *μ*M/L	Increase gene expression of *COL II* and *ACAN*	[32]
	Human UCMSCs	Activate JNK/RUNX1 pathway and suppress *β*-catenin/RUNX2 pathway	1 *μ*M/L	Elevate accumulation of extracellular matrix and chondrogenic gene expression of *SOX9*, *COL II*, and *ACAN*	[33]
	Human BMSCs	NA	100 nM/L	Increase gene expression of *SOX9*, *RUNX2*, *SERPINB2*, and *SERPINA9*	[34]
Melatonin	Human BMSCs	Attenuate IL-1*β*-induced activation of NF-*κ*B pathway	50 nM/L	Save IL-1*β*-impaired MSC chondrogenic differentiation	[41]
	Human BMSCs	NA	50 nM/L	Enhance accumulation of GAG, COL II, and COL X	[42]
CS	Goat BMSCs	NA	CS-based hydrogels	Promote MSC chondrogenic differentiation and inhibit chondrocyte hypertrophy	[45]
Ghrelin	Rat BMSCs	Enhance phosphorylation of ERK1/2 and DMNT3A	10 nM/L	Upregulate expression of *COL II*, *SOX9*, and *ACAN* and enhance accumulation of collagen and GAG *in vitro*; improve cartilage repair effect of BMSCs *in vivo*	[50]
Atractylenolides	Rat BMSCs	Activate SHH pathway	30 *μ*g/mL	Increase gene expression of *SOX9*, *COL II*, and *ACAN*	[51]
FSTL-1	Mouse MSCs	Activate TGF-*β* pathway	5 *μ*g/mL	Upregulate expression of *SOX9* and *COL II*	[53]

TGF-*β*: transforming growth factor beta; BMSCs: bone marrow-derived mesenchymal stem cells; MSCs: mesenchymal stem cells; SDSCs: synovial membrane-derived mesenchymal stem cells; BMPs: bone morphogenetic proteins; ADSCs: adipose tissue-derived mesenchymal stem cells; NA: not applicable; FGF-2: fibroblast growth factor-2; GAG: glycosaminoglycan; TSP-2: thrombospondin-2; UCBSCs: umbilical cord blood-derived mesenchymal stem cells: KGN: kartogenin; UCMSCs: umbilical cord-derived mesenchymal stem cells; COL II: type II collagen; COL X: type X collagen; CS: chondroitin sulfate; FSTL-1: follistatin-like protein-1.

**Table 2:** Effects of different physical stimulation on MSC chondrogenic differentiation.

Physical stimuli	Cell type	Mechanism	Manner	Effect	Ref.
Vibration	Rat BMSCs	Activate Wnt/*β*-catenin pathway	Low-magnitude (0.49 g) and high-frequency (40 Hz) vibration (30 min/day, 21 days)	Promote MSC chondrogenic differentiation and inhibit hypertrophic differentiation	[123]
Tensile	Rabbit BMSCs	NA	Cyclic dynamic square wave tensile at 5, 10, 15, and 20% of strain, 0.5 Hz (4 h/day, 10 days)	Improve chondrogenic phenotype of MSCs	[124]
Compression	Human BMSCs	Activate TGF-*β*/Activin/nodal pathway and suppress BMP/GDP and integrin/FAK/ ERK pathways	Cyclic dynamic compression force at 5% of strain, 1 Hz (2 h/day, 21days)	Enhance MSC chondrogenic differentiation and suppress chondrocyte hypertrophy	[125]
	Rabbit BMSCs	NA	Cyclic dynamic compression force at 10% of Strain, 1 Hz (2 h/day, 21 days)	Enhance MSC chondrogenic differentiation and suppress chondrocyte hypertrophy and fibrocartilage formation	[126]
Microgravity	Rabbit BMSCs	Suppress IHH and SHH pathways	Rotation at 12–14 rpm for 21 days	Enhance chondrogenic differentiation and attenuate chondrocyte hypertrophy and aging of MSCs	[28]
LIPUS	C3H10T1/2 cells	NA	LIPUS at 30 mW/cm^2^, 1 MHz with a pulse duration of 200 *μ*s repeated at 100 Hz (20 min/day)	Increase the expression of *COL II* and *SOX9*	[127]
	Rat BMSCs	Inhibit cell autophagy	LIPUS at 50 mW/cm^2^, on–off ratio of 20%, and irradiated with 3 MHz for 20 min (once a day, 10 days)	Increase cartilage-like ECM accumulation and gene expression of *COL II*, *SOX9*, and *ACAN*	[128]
	Rabbit BMSCs	NA	MSC-seeded PGA scaffold was subcutaneously implanted into mouse and treated with LIPUS at 200 mW/cm^2^, 0.8 Hz (10 min/day, 4 weeks)	Increase collagen and GAG content and mechanical properties of the scaffold	[129]
Electric field	Human ADSCs	NA	Electric field at 20 mv/cm, 1 kHz (20 min/day, 7 days)	Increase gene expression of *COL II* and *SOX9*; decrease gene expression of *COL I* and *COL X*	[131]
	Mouse BMSCs	Activate P2X_4_, TGF-*β*, and BMP pathways	Electrical field at 5 V/cm, 5.0 Hz with a duration of 8 ms for 3 days	Increase gene expression of *COL II*, *SOX9* and *ACAN* and accumulation of COL II and GAG	[133]
	Swine BMSCs	Downregulate the expression of DMMT1 and increase methylation of the promoters of OCT4 and NANOG	Nanosecond pulsed electrical field of 10 ns at 20 kV/cm or 100 ns at 10 kV/cm, 1 Hz for 14 days	Enhance cartilaginous ECM accumulation and gene expression of *COL II* and *SOX9*	[135]
Electromagnetic field	Human BMSCs	NA	Electromagnetic field at 5 mT, 15 Hz (45 min/8 h, 21 days)	Increase gene expression of *COL II* and GAG/DNA content	[137]
	Human BMSCs	Stimulate calcium influx	Electromagnetic field at 2 mT, 15 Hz for 10 min once on day 1 induction	Enhance cartilaginous ECM deposition and gene expression of *COL II* and *SOX9*	[138]

BMSCs: bone marrow-derived mesenchymal stem cells; MSCs: mesenchymal stem cells; NA: not applicable; LIPUS: low-intensity pulsed ultrasound; ECM: extracellular matrix; PGA: polyglycolic acid; ADSCs: adipose tissue-derived mesenchymal stem cells; COL II: type II collagen; GAG: glycosaminoglycan.

## Data Availability

The references used to support the findings of this study are included within the article.
